# Stakeholders’ views on the utility and employment strategies of clinical associates

**DOI:** 10.4102/safp.v64i1.5598

**Published:** 2022-10-28

**Authors:** Arthur Setlhapelo, Jacqueline E. Wolvaardt

**Affiliations:** 1 School of Health Systems and Public Health (SHSPH), Faculty of Health Sciences, University of Pretoria, Pretoria, South Africa

**Keywords:** clinical associates, human resources, utility, recruitment, retention

## Abstract

**Background:**

Clinical associates (ClinAs) were introduced into the South African healthcare system to increase the numbers of skilled health professionals. Little is known on how they are viewed. This study explored stakeholder views on the utility and employment strategies of ClinAs in the public sector.

**Methods:**

A mixed-methods design was used. An online survey was used to collect data from operational stakeholders, while online interviews explored strategic stakeholders’ views.

**Results:**

Forty-five operational stakeholders participated. The view of ClinAs’ contribution to the joint management of four common health conditions was strong (91% – 96%). The poorest agreement was their perceived contribution to maternal health (38%). There was a strong agreement (mean = 6.13, s.d.: 0.94) that conditions of ClinAs practice are met. Clinical associates were viewed as being able to work with others (mean = 6.11, s.d.: 0.98) and contribute to service improvement (mean = 6.47, s.d.: 0.62). There was a low agreement regarding the positive impact of recruitment (mean = 2.93, s.d.: 1.99) and retention strategies on ClinAs (mean = 2.75, s.d.: 1.51). The six key strategic stakeholders ascribed the slow progress made in career development, career progression, post creation and professional autonomy to the uncertainty regarding the scope of practice and perceived lack of support.

**Conclusion:**

The utility of ClinAs to provide health services in the public sector is clear, and their contribution is valued. The lack of progress around many of the human resource issues is a constraint that needs a champion if this cadre is to fully realise their potential.

**Contribution:**

Clinical associates are valued at service delivery level, but appear overlooked higher up.

## Introduction

The healthcare system in South Africa is currently under reform with the aim of having a healthcare system that is unified and in line with the principle of Universal Health Coverage (UHC).^1^ South Africa is therefore laying a foundation to implement a National Health Insurance (NHI), which is intended to ensure the constitutional right of all citizens to equitable access to affordable and quality healthcare services.^1^ The successful implementation of the NHI scheme is dependent on having sufficient trained healthcare professionals at the right place and time to provide the necessary healthcare services.^2,3^ These health professionals include clinical associates (ClinAs), especially in peripheral and rural district hospitals and clinics.^4^

The work of ClinAs is guided by the comprehensive healthcare approach that is embedded in the practice of family medicine and primary healthcare.^5,6^ Clinical Associates are part of the district and primary healthcare teams and work in casualty departments, medical and surgical wards, maternity and paediatric units, operating theatres and outpatient departments. They assist in providing outreach programmes at primary healthcare facilities.^5^ Clinical associates perform tasks, such as history taking, physical examination, perform and order basic investigations and interpret findings, and manage patients based on outcomes of their assessments together with the medical team. In maternity and paediatric units, ClinAs deliver and assess neonates, discuss and manage complicated cases with midwives and medical practitioners and make appropriate referrals to a higher level of care when necessary.^6,7^ Clinical associates are part of operating theatre teams and assist with caesarean section procedures and other major surgeries at district hospitals. They provide local and spinal anaesthesia under the supervision of senior medical practitioners and perform minor surgical procedures such as circumcisions and reduction of fractures and dislocations. They provide required resuscitation of patients and other basic procedures mentioned in the scope of practice and as guided by senior medical practitioners.^5,6,7^

A study done in Gauteng found that a number of the nursing departments recognised ClinAs as part of an effective medical team. Many health professionals had positive views of ClinAs, but those who did not interact with ClinAs were less likely to recognise the presence and effectiveness of ClinAs.^8^ In a case study done in Mpumalanga, some healthcare professionals were not keen to work with ClinAs despite evidence of improved access to care for clients and a reduction in the waiting times in casualty and outpatient departments.^9^

Clinical associates, together with other healthcare workers, are able to provide comprehensive healthcare to a large number of clients. A study in 2018 from the Tshwane district in South Africa found that almost 90% of circumcisions between 2014 and 2015 were done by ClinAs.^7,10^ Thus, the clinical workload of medical practitioners is reduced by sharing tasks with ClinAs.^11^

The employment of ClinAs is cost-effective without compromising the quality of healthcare, and this in turn has the potential to increase Human Resources for Health (HRH) in peripheral and rural communities.^12^ Global findings suggested that midlevel medical workers provide quality healthcare services with outcomes that are similar to medical practitioners.^13^ The effectiveness of mid-level medical workers in other countries is attributed to the ongoing support and recognition of this profession.^13^

The intention of the National Department of Health (NDOH) was to include ClinAs in the Occupation Specific Dispensation (OSD) for Medical Doctors, Dentists and Specialists in the midlevel health category, continuously develop and fund new ClinAs posts and clarify their relative position in the healthcare team.^14^ Clinical associates are categorised as midlevel health workers as they meet the criteria of shorter duration of training compared with medical practitioners, provide clinical care and engage in preventive care and health promotion.^13^ The HRH Strategy for the Health Sector 2012/13 – 2016/17 plan acknowledged ClinAs as one of the additional important tools to enhance healthcare service delivery in the public health sector. The expanded use of midlevel medical workers is one of the significant policy opportunities for South Africa to ensure the expansion of healthcare coverage to communities with little access to health services.^13^ The medium-term strategy was to ensure ClinAs presence on the government payroll system and build career pathways and employment opportunities in the health sector (private and public).^14^

However, these policies have not yet been signed into agreement with the relevant Sectoral Bargaining Council and other relevant stakeholders. This delay is reported to be because of the ongoing evaluation of the OSD by the Department of Public Service and Administration. This process may have negatively affected the determination of the ClinAs salary packages as the current salary levels were temporarily created pending review.^15,16^ Although the initial intentions of policies aimed at recruiting, training and employing ClinAs in the public health sector have been effective, the morale of ClinAs is low because of the perceived delayed support of the ClinA programme.^7,11,17,18^ Failures to create posts for ClinAs in the public sector and provide support, ongoing training and provision of adequate salaries have seen an outflux of ClinAs from the public sector into the private sector.^7^

Understanding the perceived utility of health professionals, including ClinAs, may be significant for the current and future planning of the healthcare delivery interventions. This understanding could prevent the possible loss of current ClinAs, as has been noted among other health professional categories because of the low employment of new graduates in the public sector.^19^ The first cohort of ClinAs joined the health workforce in 2011; the continued production of ClinAs has contributed to improved healthcare services provision in the public sector and has alleviated some of the workforce shortages by adding more than 1000 registered ClinAs.^20,21^ A study conducted on the opinions of medical practitioners who were supervising ClinAs in North West and Gauteng provinces had positive views on their contribution to improved patient care.^22^ The contributions of ClinAs are in line with the goals of the NHI to deliver equitable healthcare services that are efficient and effective.^7,22^

This study describes the views of key operational and strategic stakeholders on the utility and retention of ClinAs in the public sector. The refined Theoretical Domains Framework (TDF) founded on capability, opportunity and motivation was used to frame this research.^23^

Capability is a combination of knowledge, skills, behaviour and decision processes. Opportunity is a combination of environmental context and resources as well as social influences. Finally, motivation is a combination of social and professional role and identity, beliefs about capabilities and consequences, optimism, reinforcement, intentions, goals and emotions.^23^ The TDF was useful in this research as it allows for individual and contextual factors.

## Research methods and design

### Study design

This study used a sequential explanatory mixed methods design.^24^ A descriptive cross-sectional design was used to collect data on the views and knowledge and experiences of key stakeholders at the operational level who had been exposed to working with ClinAs. The next step used a descriptive qualitative study design to explore strategic key stakeholders’ perceptions on the utility of ClinAs as part of HRH strategy.

### Study setting

The study was conducted at public healthcare facilities where ClinAs are employed in the Eastern Cape, Gauteng, KwaZulu-Natal, Limpopo, Mpumalanga and North West provinces in South Africa. The facilities were determined by the provincial footprints of the three universities (the University of Pretoria, University of the Witwatersrand and Walter Sisulu University) that train ClinA students.

### Study population and sampling strategy

#### Quantitative phase

The study population for this phase were key stakeholders at the operational level in public sector healthcare facilities that employ or train ClinAs. Four participants from each training facility and operational level key stakeholders at district hospitals that employed ClinAs were invited to participate. The participants were the hospital Chief Executive Officers (CEOs), Clinical Managers, Nursing Services Managers and Human Resources Managers. Participants who had worked with ClinAs for less than a year were excluded.

#### Qualitative phase

The qualitative phase included key strategic stakeholders from the NDOH, provincial DOH departments, the professions regulatory body, tertiary institutions, department of higher education, bargaining council, professional association and trade union sectors. The key strategic stakeholder participants were selected on the basis of being able to explain the findings of the quantitative phase and articulate the current impact of ClinAs in the public sector. No exclusion criteria were used. A minimum of one individual from each group of stakeholders were invited for the interviews with the aim of achieving theoretical saturation.^25^

Non-probability sampling was used to purposely select key policy stakeholders at the strategic level who are knowledgeable about the ClinA programme. The number of interviews was guided by theoretical saturation to ensure that adequate data were collected.^25^

### Data collection

#### Quantitative phase

The tool was developed from a review of the literature, specifically on the Entrustable Professional Activities, the Regulations Defining the Scope of Practice of Clinical Associates (2016), contributions to the management of the quadruple burden of disease in South Africa, current employment strategies recommended for HRH and the Family Physician Impact Assessment Tool, which has been previously tested in the Western Cape.^26^

The questionnaire included demographic and facility details, 22 closed-ended Likert scale, multiple choice questions and three open-ended questions. Participants were invited by email to participate in the web-based Qualtrics survey. The questionnaire underwent pretesting in a pilot study to ensure that the survey questions were valid and reliable.

#### Qualitative phase

A semi-structured interview guide with five questions and prompts was based on the results of the quantitative survey. The first question explored the demographic profile of participants, while the rest were chosen to address the policy implications of the results of the quantitative data:

- Can you please tell me about your role in the utility and human resource matters of healthcare professionals in South Africa?
- What do you see as one of the major challenges in the public health system in terms of human resources for health?
- What is your opinion on the utility of clinical associates in the health system?
- What do you think are the strategic and policy challenges to recognise, employ and support clinical associates?
- What do you think is the way forward, and do you have any other thoughts and comments?

Interviews were conducted online in line with coronavirus disease 2019 (COVID-19) protocols, and data were audio recorded. Trustworthiness of the data was achieved by using multiple sources of data. The views of key stakeholders and the outcomes of the quantitative survey are described in detail in order to promote transferability. The input of external expert researchers enhanced the dependability of the research findings. The principle of confirmability, or the neutrality and accuracy of the data, was ensured by having external research experts assessing and guiding the research process and by the researcher maintaining an audit trail.

### Data analysis

#### Quantitative phase

Data from the survey were exported from Qualtrics to Stata 15 and were analysed with the assistance of a statistician. The values of the means and standard deviations (SD) were measured from the outcomes of the 7-point Likert scale ranging from 1 (strongly disagree) to 7 (strongly disagree).

#### Qualitative phase

Audio recordings were transcribed, and transcriptions were coded manually. The data were analysed inductively, and themes and categories are reported.

## Results

The results are structured to first present the results of the quantitative phase before the findings of the qualitative phase. The quantitative phase results are presented as details of participants, contributions of ClinAs, conditions of clinical associate’s practice, individual professional awareness, healthcare services and employment strategies.

### Quantitative phase

Table 1 presents details of hospitals and participants. Permission for the study included 21 hospitals, 19 of which participated while two did not respond.

**TABLE 1:** Details of participating hospitals and participants.

Variable	*n*	Position			
		CEO	CM	NSM	HRM
**Province**					
Mpumalanga	25	8	9	6	2
Eastern Cape	8	2	3	2	1
KwaZulu-Natal	7	2	2	2	1
Limpopo	3	1	1	1	-
Gauteng	2	-	-	1	1
Total	45	13	15	12	5
**Hospital category**					
Rural	35	11	12	9	3
Urban	10	2	3	3	2
Total	45	13	15	12	5
**Hospital level**					
District	42	12	13	12	5
Regional	2	1	1	-	-
Tertiary	1	-	1	-	-
Total	45	13	15	12	5

*n*, number of participants; CEO, Chief Executive Officer; CM, Clinical Manager; NSM, Nursing Services Manager; HRM, Human Resources Manager.

A total of 76 survey questionnaires were sent out to participants at 21 hospitals, with a response rate of 59% (45/76). The survey was sent out to hospitals’ CEOs, CMs, NSMs and HR Managers (HRMs) in order to incorporate a wide view of the utility of ClinAs at an operational level.

A total of 35 (78%) participants were from a rural hospital. The biggest proportion of the participants was CMs (33%) and the smallest proportion was HRMs (11%).

#### Contributions of clinical associates

Table 2 presents the perceived contribution of ClinAs to the overall management of common conditions at hospitals. There were four particular areas where over 90% of participants thought that ClinAs contributed to the overall management: HIV, non-communicable diseases, tuberculosis (TB), and injury and trauma conditions. The area where the participants scored the contributions of ClinAs the lowest (38%) was for maternal health (obstetrics and gynaecology). This item was also one where the participants were mostly unsure about the contributions of ClinAs.

**TABLE 2:** The perceived contributions of ClinAs to the overall management of a selection of common conditions.

Variable	Options	Total (*n* = 45)		Position							
		*n*	%	CEO (*n* = 13)		CM (*n* = 15)		NSM (*n* = 12)		HRM (*n* = 5)	
				*n*	%	*n*	%	*n*	%	*n*	%
HIV and opportunistic infections	Yes	43	96	12	92	14	93	12	100	5	100
	No	2	4	1	8	1	7	-	-	-	-
	Unsure	-	-	-	-	-	-	-	-	-	-
Non-communicable (hypertension, diabetes, mental health conditions, etc.)	Yes	43	96	12	92	14	93	12	100	5	100
	No	2	4	1	8	1	7	-	-	-	-
	Unsure	-	-	-	-	-	-	-	-	-	-
Tuberculosis (TB)	Yes	42	95	13	100	14	93	11	92	4	100
	No	2	5	-	-	1	7	1	8	-	-
	Unsure	-	-	-	-	-	-	-	-	-	-
Injury and trauma (assault, accidents, etc.)	Yes	41	91	12	92	14	93	10	83	5	100
	No	3	7	1	8	1	7	2	17	-	-
	Unsure	1	2	-	-	-	-	-	-	-	-
Coronavirus disease 2019 (COVID-19)	Yes	35	78	10	77	11	73	11	92	3	60
	No	8	18	3	23	4	27	1	8	2	40
	Unsure	2	4	-	-	-	-	-	-	-	-
Paediatric and child health	Yes	32	71	11	86	9	60	10	83	2	40
	No	7	16	1	7	5	33	2	17	1	20
	Unsure	6	13	1	7	1	7	-	-	2	40
Assisting with surgical cases like C-sections, laparotomy, tubal ligation and other routine surgeries	Yes	32	71	10	77	13	86	8	67	1	20
	No	8	18	3	23	1	7	3	25	1	20
	Unsure	5	11	-	-	1	7	1	8	3	60
Maternal health (obstetrics and gynaecology)	Yes	17	38	6	46	6	40	5	42	2	40
	No	16	35	1	8	8	53	5	42	3	60
	Unsure	12	27	6	46	1	7	2	17	-	-

*n*, number of participants; CEO, Chief Executive Office; CM, Clinical Manager; NSM, Nursing Services Manager; HRM, Human Resources Manager.

#### Conditions of clinical associate’s practice

Participants strongly agreed that ClinAs practised under the direct and/or indirect supervision of medical practitioners at their hospitals (mean = 6.13, standard deviation [s.d.]: 0.94). Participants agreed that ClinAs identify themselves as such to co-workers and patients (mean = 5.53, s.d.: 1.49). Participants also agreed that ClinAs do not act as locum tenens for medical practitioners (mean = 5.33, s.d.: 1.65).

#### Individual professional awareness

Participants strongly agreed that the practice of ClinAs is ethically guided (mean = 6.13, s.d.: 0.79), demonstrated professionalism (mean = 5.98, s.d.: 1.03) and concurred that ClinAs engaged in continuous learning (mean = 5.73, s.d.: 1.17).

#### Healthcare services and employment strategies

Table 3 shows participants’ opinions on the contributions of ClinAs to working in a team and to services offered by the hospital. The participants strongly agreed that ClinAs are able to work with other healthcare workers and contribute to the delivery of hospital healthcare services. The highest overall mean (6.11, s.d.: 0.98) was for working for others in preventative healthcare, and the lowest overall mean (5.75, s.d.: 1.26) was for understanding their scope of practice.

**TABLE 3:** Results on the contribution of ClinAs to teamwork and the services provided by the hospital.

Domains statements	All (*n* = 45)		CEO (*n* = 13)		CM (*n* = 15)		NSM (*n* = 12)		HRM (*n* = 5)	
	Mean	s.d.	Mean	s.d.	Mean	s.d.	Mean	s.d.	Mean	s.d.
**Working with others**										
Clinical associates are able to team up and work with medical practitioners, professional nurses and other important practitioners within the district healthcare services, with the focus on preventative healthcare	6.11	0.98	6.46	0.51	5.87	1.46	6.17	0.72	5.80	0.45
Clinical associates are able to perform any act delegated to him or her by the supervising medical practitioner in accordance with their level of experience and training	5.98	0.99	6.23	0.72	5.67	1.40	6.25	0.62	5.60	0.55
Clinical associates know and understand the limitations of their scope of practice (i.e. knows when to refer or ask for help appropriately)	5.75	1.26	5.85	0.90	5.87	1.30	5.92	1.08	4.80	2.17
**Contributing to the hospital**										
Clinical associates have assisted with the patients’ experience of care at the facility, that is, try to reduce waiting times, etc.	6.53	0.59	6.54	0.52	6.53	0.64	6.58	0.67	6.40	0.55
Clinical associates have contributed jointly with other health professionals in improving the quality of healthcare services at your facility	6.47	0.62	6.54	0.52	6.27	0.60	6.83	0.58	6.00	0.71
The recruitment of more clinical associates will assist with having more health workforce with the required medical knowledge and clinical skills at your facility	6.33	1.65	6.85	0.37	6.07	1.91	5.83	2.21	7.00	0.00
Clinical associates understand how the healthcare system operates	6.20	0.59	6.15	0.55	6.20	0.56	6.33	0.65	6.00	0.70

CEO, Chief Executive Office; CM, Clinical Manager; NSM, Nursing Services Manager; HRM, Human Resources Manager.

Table 4 shows participants’ opinions regarding the recruitment and retention strategies for ClinAs. Participants were neutral on the creation of awareness for the ClinAs as the new profession at their hospitals. Participants disagreed that there has been ongoing creation of posts for ClinAs at their hospitals. Regarding retention strategies: participants were neutral on the scope of practice providing room for professional autonomy and the availability of CPD activities for ClinAs. The participants further disagreed that there have been adequate retention strategies for ClinAs at their hospitals.

**TABLE 4:** Results on employment strategies.

Domains statements	All (*n* = 45)		CEO (*n* = 13)		CM (*n* = 15)		NSM (*n* = 12)		HRM (*n* = 5)	
	Mean	s.d.	Mean	s.d.	Mean	s.d.	Mean	s.d.	Mean	s.d.
**Recruitment strategies**										
The government has put measures in place and supported efforts to create the needed awareness for clinical associates as the new profession at your facility	4.02	1.92	4.08	1.93	3.07	1.62	5.08	1.88	4.20	1.79
The provincial human resource planning department is continuously creating, funding, and advertising posts for clinical associates at your facility	3.42	1.80	3.60	1.89	2.67	1.50	4.00	2.09	3.80	1.30
The government has been able to provide your facility with the required number of clinical associates to increase the health workforce	3.24	1.65	3.69	2.06	2.93	1.90	3.42	1.91	2.60	1.34
There has been a significant gradual increase in clinical associates’ permanent employment at your facility	2.93	1.99	3.61	2.26	2.47	1.96	3.00	2.00	2.40	1.14
**Retention strategies**										
The scope of practice for clinical associates provides room for professional autonomy	4.80	1.50	5.23	1.48	4.40	1.50	4.75	1.81	5.00	0.00
There has been the provision of strategies for continuous professional development activities that support clinical associates	4.40	1.93	5.00	1.87	3.73	2.05	4.75	1.91	4.00	1.58
There has been government’s advocacy for professional support of clinical associates	3.95	1.94	4.61	2.10	2.93	1.71	4.67	1.67	3.60	1.82
The government has provided clinical associates at your facility with opportunities for career development and promotion to higher notches and posts	2.75	1.51	2.77	1.30	2.07	1.28	3.50	1.83	3.00	1.22

CEO, Chief Executive Office; CM, Clinical Manager; NSM, Nursing Services Manager; HRM, Human Resources Manager.

**FIGURE 1:**
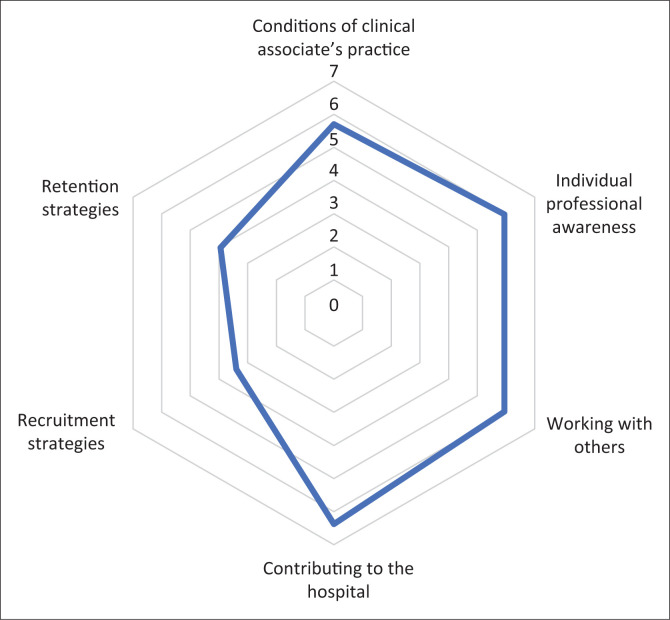
The perceived overall impact of clinical associate’s utility and employment strategies.

Figure 1 plots the six domains of the quantitative phase in a radar graph. The points on the graph are the mean values of the domains. The radar graph shows the high positive utility impact of ClinAs on service delivery within hospitals, followed by their ability to work with others, and having individual professional awareness. The radar graph also shows that participants view ClinAs as meeting their conditions of professional practice. There is, however, low positive agreement for both the impact of the recruitment and the retention strategies on the utility of ClinAs.

## Qualitative phase

Six of the 12 key strategic stakeholder participants invited for the in-depth interviews participated in the study. The interviewees were from the Department of Health, health professions regulatory bodies and tertiary institutions.

Four themes emerged from the data, namely:

- the scope of practice and professional autonomy;
- career development and progression;
- posts for clinical associates; and
- continuous professional development activities.

### The scope of practice and professional autonomy

Interviewees agreed that the current scope of practice provides for increased professional autonomy over time. The approach of supervised practice was reported to be protective from medico-legal challenges, and experiences from other countries support this notion of supervised practice. The increasing levels of trust over time between ClinAs and medical practitioners have shown to lead to less need for direct supervision. Interviewees reported ongoing proposals submitted regarding this shift in professional autonomy of more experienced ClinAs. These proposals are guided by the current scope of practice that allows for ClinAs’ increased independent decision-making regarding patient care:

‘The professional autonomy may be limited by the HPCSA’s registration of ClinAs under the category of “Supervised Practice”. In this instance it means the professional autonomy is guided by the supervision of medical practitioners, and within that supervision, clinical associates may have to find a way to function. However, the ClinAs scope of practice speaks about the levels of supervision based on experience and years of practice. But there has not been enough campaigning and emphasise for dealing with concerns around the professional autonomy of ClinAs.’ (Key strategic stakeholder, academic, lecturer, #2)

Interviewees were cognisant that the requirement of direct supervision is dependent on adequate numbers of medical practitioners in these settings, and the overall constraints of ensuring filled medical practitioner posts have a knock-on effect for the ClinAs:

‘Some ClinAs in hospitals in the province are working independently with indirect supervision after being under direct supervision as mentioned in the scope of practice. This is after the development of trust between the medical practitioners and ClinAs, and their ability to prove to the medical practitioners that they can work with them and alone at times.’ (Key strategic stakeholder, academic, lecturer, #1)

Interviewees argued for some form of ClinAs’ independent practice following clinical clerkships.

### Career development and progression

Interviewees were of the opinion that the high clinical workload was one of the driving factors for the creation of the ClinA programme. The reported high clinical workload extends throughout the various specialist disciplines; however, there has not been a strategy of preparing ClinAs for work in these specialist areas, and only one postgraduate course in Emergency Medicine is available:

‘This is a real challenge we are facing now because there are currently no opportunities for career paths right from the start [*year 201 1*] of the Clinical Associate Programme. We need to make time to remedy this matter.’ (Key strategic stakeholder, academic, lecturer, #1)

There are other general postgraduate courses for healthcare workers, with good uptake by ClinAs. Interviewees thought that more postgraduate courses that match the burden of disease were needed, but that progress in registering additional qualifications for ClinAs with the Health Professions Council of South Africa is slow.

The absence of posts in the public healthcare sector for ClinAs with postgraduate qualifications was raised as a concern by interviewees. The slow progress made in having additional qualifications for ClinAs, the lack of professional growth opportunities, career pathing opportunities and salary progression were raised by the interviewees as substantive challenges:

‘It is evident that there is an urgent need for career pathing to grow in terms of specialization and management posts for ClinAs in the public health sector instead of the private due to the high number of communities that need continuous access to public healthcare services.’ (Key stakeholder, academic, lecturer, #2)

### Posts for clinical associates

Interviewees explained that ClinA programmes are effective in settings similar to South Africa, and while there was no doubt that the programme has good intensions and that there is evidence that routine clinical workload can be shared with this cadre, there has not been a significant increase of ClinAs posts in the public sector:

‘Vacancies for clinical associates are not being regularly generated, funded and advertised like other healthcare professions vacancies. This might be because there has not been a significant push from people who create vacancies, and those who want vacancies to be created.’ (Key strategic stakeholder, public sector, head of department, #5)

The current challenges with the creation of posts for ClinAs were perceived to be because of the lack of advocacy for the creation of posts and uncertainty of professional identity. The implementation of the ClinA programme may have been hampered by not addressing these aspects at inception. This oversight therefore led to delays regarding the categorising of ClinAs according to years of practice, lack of career pathing opportunities, absence of formal report on services of ClinAs and general loss of follow-up on career development and progression initiatives.

### Continuous professional development activities

Career development and progression rely in some part on continuous professional development (CPD). Interviewees reflected that there are no organisations that offer CPD activities specifically for ClinAs:

‘Clinical associates need to have a certain number of hours of CPDs just like other health professions. But there is no entity that is designated with officially providing those CPD activities. That appears like a loose structure in terms of how ClinAs can go about in getting CPDs. If there was to be an organization that will take upon the structuring, setting up, and processing of CPD activities for ClinAs, there would a much better job of having CPDs available.’ (Key strategic stakeholder, academic, lecturer, #3)

Interviewees reflected that teaching hospitals have more general CPD activities compared with other hospitals, which in turn deepens the inequity of growth opportunities.

## Discussion

The study explored and described the views of the key operational and strategic stakeholders on the current utility and employment strategies of ClinAs in selected public sector hospitals. Operational stakeholders who had been exposed to the work of ClinAs were able to share their experiences of having ClinAs at their hospitals. Strategic stakeholders expanded on the results of the survey in in-depth interviews.

Clinical associates were reported to have contributed to the management of the majority of common health conditions at hospitals. These include more than 90% of participants who agreed that ClinAs have contributed to the overall management of health conditions such as HIV, non-communicable diseases and trauma. The contributions of ClinAs to other overall management health conditions like COVID-19, paediatric and child health, and assisting in surgery were acknowledged. These findings are important, particularly in a country that is faced with a quadruple burden of disease. The contributions of ClinAs in this study are similar to a study conducted in Kenya where the work of cadres like ClinAs was seen as important in providing healthcare services of expected quality, particularly in district hospitals and rural areas.^27^ In this study, the perceived contributions to the management of specialised maternal health conditions were relatively poor and may be because of the need for a further specialised training of ClinAs in these specialities.^28^

Participants in this study agreed that conditions of practice for ClinAs and the requirements for their individual professional awareness had been met as they are required by the health professions regulatory body and are stipulated in their scope of practice. The strong agreement on the practice of ClinAs that is supervised by medical practitioners indicates the adherence of ClinAs to the regulations that specify the functions of their scope of practice. The agreement by participants on the practice of ClinAs that shows professionalism and being ethically guided further illustrates adherence of ClinAs to ethical guidelines for good practice in the healthcare professions. The finding that the conditions of practice for ClinAs and their individual professional awareness are satisfactory could be attributed to the HPCSA who plays a significant role to remind health practitioners of the ethical rules, which emphasise that practitioners may not perform any duties that are outside their scope of practice.^29^

The ability of healthcare workers to work as a team protects patients from health risks and improves health outcomes. Teamwork further creates a peaceful working environment for healthcare workers.^30^ Health facilities with reported high levels of clear roles for each healthcare worker and management who is mindful of interdependence have lower rates of injuries and illnesses on duty and low turnover of healthcare workers.^31^ Participants in this study reported positively on the ability of ClinAs to work with other healthcare workers, perform any act delegated to them and their understanding of their scope of practice. These outcomes indicate the contributions of ClinAs to teamwork and a holistic approach to the delivery of healthcare services.

Participants strongly agreed that ClinAs assist with patients’ experience of care at hospitals, join other healthcare workers in improving the quality of healthcare services and understand how the health system operates. The findings support the outcomes of the study conducted at a district hospital in Mpumalanga that looked into the introduction of ClinAs at their facility, which found that ClinAs assisted the hospital in maintaining and improving patient care.^9^ The strong agreement among participants that more ClinAs will strengthen their hospitals’ ability to provide the needed quality of healthcare services is evidence of the high regard for their contribution. The efficient use of HRH that include more ClinAs would assist an overburdened public sector, particularly during the COVID-19 pandemic.

The study did not find strong agreement on the provision of putting measures in place or supported efforts to create the needed awareness for ClinAs as the new profession at hospitals. Other findings with low mean values were on the reported slow progress made in the continuous creation, funding and advertisement of posts for ClinAs at hospitals. The interviewees explained that this may be because of a lack of advocacy for the ClinA profession and uncertainty around the profession’s identity. Similar outcomes were found in a study that explored the success and challenges of cadres like ClinAs in other countries. Lack of awareness and acceptance of these cadres in the healthcare system were found to be some of the delaying factors for the successful running of the programmes similar to that of ClinAs.^32^ The need to increase awareness and acceptance of ClinAs in the health system is required to show support for the profession. In published peer-reviewed studies from three continents, the employment of these cadres was found to be cost-effective in terms of their training and labour costs while providing the expected care outcomes of healthcare workers.^33^

The scope of practice for ClinAs was found by operational stakeholders not to provide room for professional autonomy, and the strategic stakeholders linked that aspect to the design of the scope of practice of ClinAs, which categorised them under ‘Supervised Practice’ at the HPCSA. Although the current scope of practice reduces the need for direct supervision of ClinAs by medical practitioners, the category of ClinAs under ‘Supervised Practice’ remains the same. Cadres like ClinAs in Rwanda were found to have been trained in having professional autonomy and function competently in their diagnostic practices.^34^ These findings support the scope of practice for ClinAs that allows independent decision-making in patient care. It is, however, significant to appreciate the protective nature of ClinAs practicing under direct and indirect supervision of medical practitioners as emphasised by some of the strategic key stakeholders in this study, and this requirement may play a crucial role around the considerations of expanding the professional autonomy of ClinAs. The proposals made by some key strategic stakeholders on the clinical clerkship programme for ClinAs similar to the internship programme for medical practitioners and pharmacists may add value to further reduce the ongoing need of supervision for ClinAs by medical practitioners and lead to some form of interdependence as its effectiveness was proven in a study conducted on community service medical practitioners.^35^

The outcome of the operational level stakeholders survey showed that the current recruitment strategies did not provide ClinAs with opportunities for career development and promotion to higher posts and salary notches as they were originally planned in 2011. The acknowledgment of key strategic stakeholders on the perceived delay in developing and implementing a strategy for ClinAs career pathing opportunities was seen as a challenge that needs to be addressed. Although there is currently one postgraduate course in Emergency Medicine specifically for ClinAs, the absence of posts in the health sector for ClinAs with postgraduate qualifications was noted as one of the reasons for the slow progress made in developing more specialist courses for ClinAs. Studies conducted in neighbouring countries of South Africa found that professional recognition, suitable employment options, opportunities for further studies and career paths that follow a multi-sector approach are significant in supporting cadres like ClinAs in healthcare facilities where they are needed most.^36,37,38^ The creation of posts for ClinAs with postgraduate qualifications and their employment in these specialist posts are key to having more career paths opportunities for ClinAs, promoting them to higher posts and retaining them in the health system, particularly the public sector.

Stakeholders at the operational level of hospitals indicated low and neutral levels of agreement on the provision of CPD activities that are inclusive of ClinAs. Although there are existing limited providers of general CPD activities for healthcare workers, this outcome highlights the need to have more CPD activities that support ClinAs. In countries where health systems are challenged by shortages of HRH and high burdens of disease, efforts are constantly required to increase HRH and strengthen their capacity to ensure that all healthcare workers are fit for purpose and practice.^39,40,41^ The reported absence of an entity by strategic stakeholders that is designated and equipped with educational resources is one stumbling block that hinders the provision of CPD activities specifically for ClinAs. Ongoing education to maintain core competencies of ClinAs and update their practices in a workplace is required, as this will ensure that they meet the goals of their healthcare facilities and the healthcare needs of communities they serve. These goals can be achieved by having online CPD activities that are responsive to the limited resources of healthcare settings where ClinAs work. This approach is supported by a study that showed acceptance of online CPD opportunities among a diverse group of healthcare workers in sub-Saharan Africa.^41^

### Strengths and limitations

The strengths of the research were the use of the study design that was able to understand the outcome of the quantitative phase, which was further explained in the findings of the qualitative phase. The findings of this study are likely to be similar to other hospitals with comparable levels of care, staffing and needs of healthcare seekers. Because of the purposive sampling of hospitals, the quantitative phase results may not be representative of the rest of the hospitals in South Africa that employ ClinAs.

The views of key stakeholders at the operational level differ according to their levels of exposure to their observed utility and employment strategies of ClinAs and that may be because of their respective roles that relate differently to ClinAs.

The study was conducted during the COVID-19 pandemic; therefore, the use of remote data collection may have lowered the response rate of participants and interviewees, as some of the individuals did not respond to the invite to participate in the study and/or were not available.

### Recommendations and implications

The study outcome described and showed the positive views of the utility of clinical associates in the healthcare system. The low-positive agreement regarding employment strategies of ClinAs suggests the need to develop, adopt and implement additional strategies and policies that support the recruitment and retention of clinical associates in the health system. The implications of the findings are to increase the number of universities that offer the degree, develop a 1-year internship and a 1-year community service programme, develop postgraduate qualifications opportunities, develop CPD opportunities and create recruitment and retention strategies for ClinAs. Further research studies could explore possible areas of specialisation for this cadre.

## Conclusion

The utility of ClinAs to provide health services in the public sector is clear, and their contribution is valued by operational stakeholders. Operational stakeholders are aware of the challenges that ClinA face, which in turn could potentially affect the retention of this cadre in their hospitals. The findings support the need to recognise and support the professional growth of ClinAs and increase their deployment in the healthcare sector, with the aim of enhancing the gains made in the provision of required quality healthcare services to communities. Strategic stakeholders were similarly aware of the challenges that hinder both the potential utility and retention of ClinAs. The lack of progress around many of the human resource issues – including the 10-year delay in including this cadre in the OSD – is a constraint that needs a champion if this cadre is to fully realise their potential.
